# Differential protein expression following low temperature culture of suspension CHO-K1 cells

**DOI:** 10.1186/1472-6750-8-42

**Published:** 2008-04-22

**Authors:** Niraj Kumar, Patrick Gammell, Paula Meleady, Michael Henry, Martin Clynes

**Affiliations:** 1 National Institute for Cellular Biotechnology, Dublin City University, Glasnevin, Dublin 9, Ireland

## Abstract

**Background:**

To ensure maximal productivity of recombinant proteins (rP) during production culture it is typical to encourage an initial phase of rapid cell proliferation to achieve high biomass followed by a stationary phase where cellular energies are directed towards production of rP. During many such biphasic cultures, the initial phase of rapid cell growth at 37°C is followed by a growth arrest phase induced through reduction of the culture temperature. Low temperature induced growth arrest is associated with many positive phenotypes including increased productivity, sustained viability and an extended production phase, although the mechanisms regulating these phenotypes during mild hypothermia are poorly understood.

**Results:**

In this study differential protein expression in suspension CHO-K1 cells was investigated following a reduction of the culture temperature from 37°C to 31°C in comparison to standard batch culture maintained at 37°C using 2D-DIGE (Fluorescence 2-D Difference Gel Electrophoresis) and mass spectrometry (MS). There is only limited proteomic analysis of suspension-grown CHO cells describing a direct comparison of temperature shifted versus non-temperature shifted cultures using 2D-DIGE. This investigation has enabled the identification of temperature-dependent as well as temperature-independent proteomic changes. 201 proteins were observed as differentially expressed following temperature shift, of which 118 were up regulated. Of the 53 proteins identified by MALDI-ToF MS, 23 were specifically differentially expressed upon reduction of the culture temperature and were found related to a variety of cellular functions such as regulation of growth (HNRPC), cap-independent translation (EIF4A), apoptosis (importin-α), the cytoskeleton (vimentin) and glycoprotein quality control (alpha glucosidase 2).

**Conclusion:**

These results indicate the extent of the temperature response in CHO-K1 cells and suggest a number of key regulatory proteins and pathways that are involved in modulating the response of cells to mild hypothermia. Regulation of these identified proteins and pathways could be useful for future approaches to engineer CHO cells for improved recombinant protein production.

## Background

Since the introduction of human tissue plasminogen activator (tPA) as the first recombinant therapeutic protein product from mammalian cells to get approval in 1986, approximately 60–70% of all recombinant proteins are being produced in mammalian cell lines [1], predominantly in Chinese Hamster Ovary (CHO) cells. The increasing demands for biologicals necessitates improved productivity from CHO cells and the methods currently applied include optimization of culture conditions (temperature, pH, aeration rate, etc.), improved reactor designs, medium formulation and cell engineering. Despite this, however, many aspects of CHO cell biology remain poorly understood.

Many culture processes operate a biphasic culture whereby cells are grown at 37°C to maximise biomass and then the culture is shifted to a lower temperature (28–33°C) to encourage protein production while maintaining a longer and more viable stationary/production phase [2-6]. Low temperature cultivation of mammalian cells is an established method to maintain cell viability while at the same time inhibiting growth; this inhibition is associated with G1-phase cell cycle arrest which has been positively correlated with increased productivity [2-9]. However, the effect of low temperature on recombinant protein productivity can be product, clone or cell line specific [10]. Increased levels of mRNA coding for the recombinant protein, either due to enhanced transcription or increased mRNA stability of the recombinant gene of interest, have also been observed during low temperature culture and also correlate with improved recombinant protein productivity [3,8]. Post-translational modifications, particularly glycosylation, which are important for biological activity of recombinant proteins, are maintained or even improved at low temperature [3,4]. In addition to affecting productivity, low temperature culture has been shown to reduce cellular metabolism (glucose/medium consumption and oxygen uptake), waste accumulation (lactate and ammonia production) and apoptosis [7,8,11].

The molecular mechanisms regulating sub-physiological temperature response of cells in culture are still poorly understood, despite a number of recent transcriptomic and proteomic profiling studies [2,12-14]. It is generally accepted that global cap-dependent protein translation decreases at sub-physiological temperatures, however IRES mediated cap-independent protein translation is active [15,16]. More than 20 proteins including cold-inducible RNA binding protein (CIRP), RNA binding motif protein 3 (RBM3), protein disulphide isomerase (PDI), nuclear localization sequence binding protein (NSR1), vimentin, phosphoglycerate kinase and heat shock cognate 71 kDa protein have been reported as being up regulated during low temperature culture [12,13,17,18]. Of these proteins, CRIP and RBM3 have been studied extensively [15,17-20]. Both CIRP and RBM3 are involved in modulation of transcription and translation by functioning as RNA chaperones [18-20]. CRIP improves recombinant protein productivity and can arrest cell growth in a cell line-specific manner whereas RBM3 facilitates cap-independent protein synthesis at low temperature [15,17,20]. RBM3 is also reported to interact with and partially block the activity of miRNAs at low temperature [19]. Altered miRNA expression following temperature shift has recently been reported and may be an important component of regulating protein expression and cell behavior at low temperature [13].

In this study, we have used two-dimensional difference gel electrophoresis (2D-DIGE) to investigate the proteomic changes associated with the shift to low temperature in a biphasic batch culture of suspension-adapted CHO-K1 cells. In this manner we wished to gain insights into the effects of low temperature culture on mammalian cells, with potential relevance to mammalian cell-based recombinant protein production.

## Results

### Cell Culture

**Figure 1:**
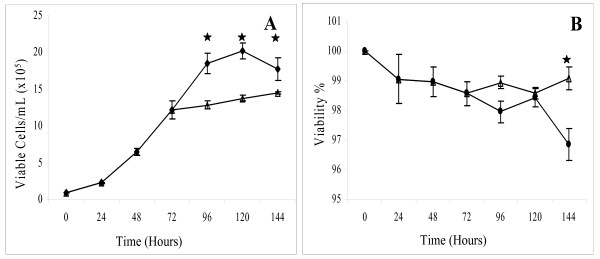
**Comparison of the viable cell number (A) and percentage viability (B) of CHO-K1 cells cultured using either a biphasic temperature shifted culture (--◆--) or using a standard culture at 37°C (--▲--). **Error bars represent the standard deviation calculated from three biological replicate cultures. '*' indicates statistically significant (p-value ≤ 0.02) differences in cell growth and viability.

Suspension-adapted CHO-K1 cells were seeded at 1 × 10^5 ^cells/mL in spinner flasks and maintained at 37°C for 144 hrs or for 72 hrs at 37°C followed by a temperature shift to 31°C for a further 72 hrs. 72 hrs was chosen for this temperature shift because it represents the mid-exponential phase of cell growth. 31°C was selected for the temperature shift as it represents the average temperature employed by others performing similar studies, i.e. 28–33°C [2,4,6,9,10,13]. As can be seen in Figure 1 the cells that were temperature shifted immediately ceased logarithmic growth and did not exceed a peak viable cell density of 1.45 × 10^6 ^± 0.013 cells/mL, whereas the cells maintained in standard culture at 37°C continued in logarithmic growth for a further 48 hrs and achieved a peak viable density of 2.02 × 10^6 ^± 0.11 cells/mL. The cells that were temperature shifted displayed a steady if slightly increasing viable cell density throughout, whereas the cells cultured at 37°C had entered the late stationary/death phase of the growth cycle by 144 hrs (Figure 1A&B). Cultures were sampled at 72 hrs and 144 hrs for protein extraction from 3 independent experiments.

### Differential analysis of proteins

**Figure 2:**
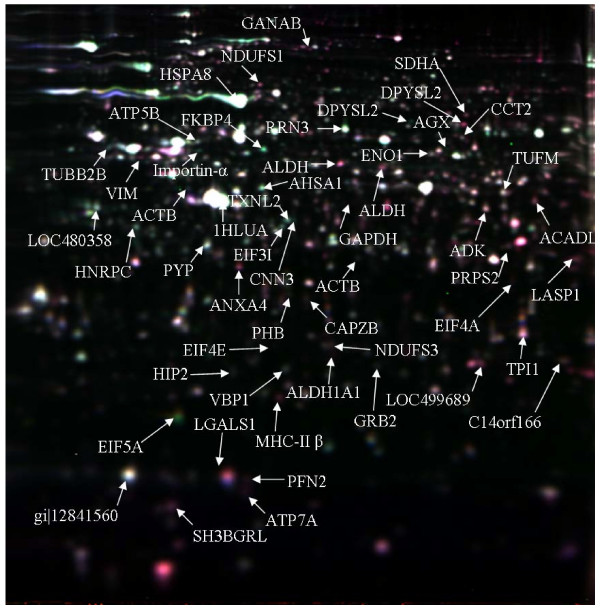
**Representative overlapping 2D-DIGE expression map of CHO-K1 proteins labelled with fluorescent dyes (Cy2, Cy3 and Cy5).** The location of differentially expressed proteins identified using MALDI-ToF MS are indicated by arrows.

2D-DIGE protein expression maps (PEMs) for the biological triplicate samples were generated for both temperature shifted and standard cultures for CHO-K1 cells at 72 hrs and 144 hrs. The 72 hrs PEMs were then compared to that at 144 hrs using the Biological Variance Analysis (BVA) module of DeCyder software. This was aided by the use of a pooled internal standard which facilitated spot matching and relative spot quantitation while minimizing the gel-to-gel variations. Spots originating from dust particles and gel impurities in each gel image were checked and removed manually to improve the quantitation and matching process. Landmarks, which aid the gel-to-gel spot matching process, were also defined in the gel to increase the accuracy of the matching algorithm. Manual checks were carried out to allow for cases where spots were detected more than once (due to spot splits by the spot detection algorithms), these spots were then merged back to improve spot matching. After spot matching and filtering, 2852 and 2885 spots were detected for the temperature shifted and standard culture experiments respectively. Spots that were 1.5 fold up/down regulated at 144 hrs compared to 72 hrs with a t-test score of ≤ 0.05 and present on all gels were considered differentially expressed (DE). A total of 201 DE spots (118 up regulated and 83 down regulated) were observed in the temperature shift experiment and 404 DE spots (210 up regulated and 194 down regulated) were observed in the standard culture experiment (Figure 2).

### Spot Identification

**Figure 3:**
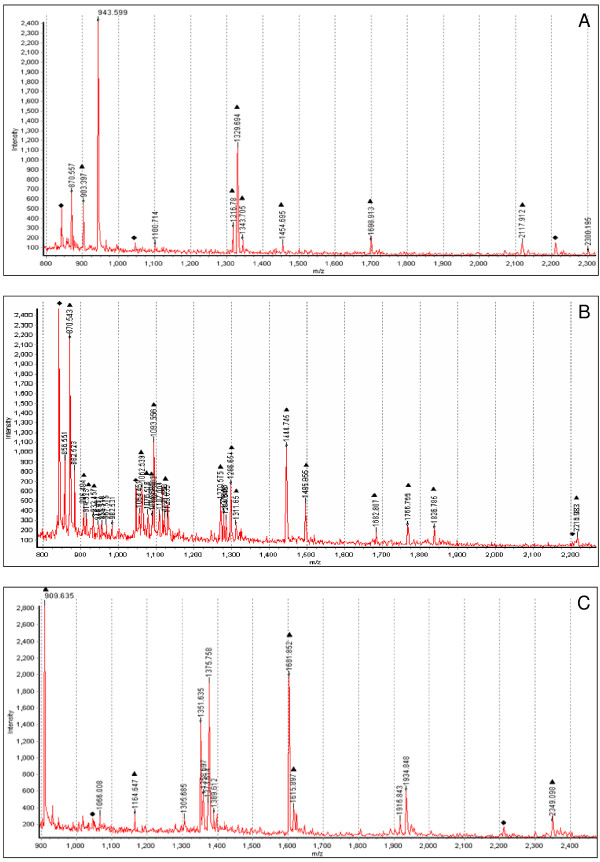
**MALDI-ToF generated spectra of Heterogeneous nuclear ribonucleoprotein C (HNRPC) (A), Vimentin (VIM) (B) and Glyceraldehyde-3-phosphate dehydrogenase (GAPDH) (C).** Trypsin digested peptides were used for identification with MALDI-ToF. Trypsin gives peaks at specific molecular weight (842.51, 1045.5 and 2211.1 m/z) and can be used as calibrants in the MS spectra. Peaks other than trypsin are from target proteins. Trypsin calibration peaks are represented by ◆ and peaks belonging to identified proteins by ▲.

Following spot picking and tryptic digestion, protein identification was carried out using MALDI-ToF MS. All identified proteins had an expectation value of 0.01 or better. Expectation value for proteins was determined by Ettan MALDI-ToF Pro evaluation software (GE Healthcare) using the Profound database search engine for peptide mass fingerprints. An expectation value of 0.01 or better means that there is ≤ 1% chance that the identification is random. Although many spots resulted in high quality spectra, they were not identified which may be due to insufficient amounts of protein, modification and/or variation of the amino acid sequences in CHO proteins or the paucity of CHO proteins in the protein databases. Despite this 53 proteins were identified (Additional file 1) and the representative spectra of 3 of these proteins (vimentin, HNRPC and GAPDH) are presented in Figure 3. Following identification of the 53 spots, all identified proteins were compared for differential expression in both temperature shifted and standard culture experiments to identify temperature-dependent and temperature-independent protein changes (Additional file 1). For example, GANAB, a glycan-processing enzyme that is involved in glycoprotein quality control in the endoplasmic reticulum [21], was 2.19 fold up regulated at 144 hrs compared to 72 hrs following temperature shift while it was unchanged when cells were maintained at 37°C. Activator of 90 kDa heat shock protein ATPase homolog 1 (AHSA1) however, was differentially expressed in both experiments (1.69 and 1.73 fold down regulated at 144 hrs following temperature shift and standard culture respectively), hence could be considered a temperature-independent protein change. A number of the identified proteins including vimentin and GAPDH have been previously identified as differentially expressed following temperature shift (Table 1). The identified differentially expressed proteins were also compared between both temperature shifted and standard cultures at 144 hrs (Table 2 &3). Three proteins, ACTB, ALDH and DPYSL2 were identified at more than one location on the gel possibly indicating some form of post-translational modification (PTM), of which PTMs in ALDH and DPYSL2 were specific to the temperature shift to 31°C. The identified proteins could be linked to a broad range of biological functions including structural, metabolic, differentiation, secretory pathways, translation, transcription, protein binding, signal transduction, cell adhesion and apoptosis.

**Table 1:** Comparison of differentially expressed proteins identified in this study with previous studies on the effect of temperature.

**Protein**	**This Study**	**Previous Study**			**Function**
			
		**Author**	**Protein/Transcript**	**Effect**	
Vimentin	Up regulated	Baik et al, 2006	Vimentin	Up regulated	Cell size, proliferation, cell growth rate and apoptosis.
GAPDH	Up regulated	Baik et al, 2006	GAPDH	Up regulated	Apoptosis, nuclear tRNA export, DNA replication, DNA repair and transcription
LGALS1	Up regulated	Baik et al, 2006	Galectin 1	No change	Cell adhesion and proliferation
ACTB	No Change	Baik et al, 2006	B-actin (Identified at multiple spots)	Up regulated at 2 spots and down regulated at 1 spot	Cell size, shape and proliferation, transcription and apoptosis
PHB	No Change	Baik et al, 2006	Prohibitin	Up regulated	Proliferation, differentiation and apoptosis
TPI1	No Change	Baik et al, 2006	Triosephosphate isomerise	No change	Stress conditions and cell death

**Table 2:** Differential expression of process-dependent proteins at 144 hrs between temperature shifted and standard culture conditions.

**Number**	**Protein Description**	**Fold Change at temperature shift (72 hrs vs. 144 hrs)**	**Fold Change at standard culture (72 hrs vs. 144 hrs)**	**Fold Change at 144 hrs (144 hrs at 31°C vs. 144 hrs at 37°C)**
1	Calponin 3, acidic	-1.96	-3.37	1.72
2	Chain A, Structure Of Bovine Beta-Actin-Profilin Complex (1HLUA)	-1.59	-1.53	-1.04
3	PREDICTED: similar to NADH dehydrogenase (ubiquinone) Fe-S protein 1, 75 kDa precursor	2	1.59	1.26
4	Succinate dehydrogenase flavoprotein subunit	1.7	1.87	-1.1
5	PREDICTED: similar to Dihydropyrimidinase related protein-2	2.58	1.97	1.31
6	Eukaryotic translation initiation factor 3, subunit I	-1.72	-2	1.16
7	Eukaryotic translation initiation factor 5A	-1.81	-2.07	1.14
8	PREDICTED: similar to Activator of 90 kDa heat shock protein ATPase homolog 1	-1.69	-1.73	1.02
9	Thioredoxin-like 2	-1.8	-1.8	1
10	Menkes disease gene product	1.79	1.6	1.12
11	FK506 binding protein 4	-2.5	-2.43	-1.02
12	PREDICTED: similar to heat shock protein 8 isoform 3	-1.9	-2.09	1.1

**Table 3:** Differential expression of proteins at 144 hrs between temperature shifted and standard culture conditions.

**Number**	**Protein Description**	**Fold Change at 144 hrs (144 hrs at 31°C vs. 144 hrs at 37°C)**
1	Vimentin	1.9
2	Calponin 3, acidic	1.72
3	Beta actin	2.2
4	Capping protein (actin filament) muscle Z-line, beta	1.88
5	Tubulin T beta15	1.76
6	Aldehyde dehydrogenase family 1, subfamily A1	1.76
7	Glyceraldehyde-3-phosphate dehydrogenase	1.64
8	PREDICTED: similar to UDP-N-acetylhexosamine pyrophosphorylase (Antigen X)	-2.90
9	Acetyl-Coenzyme A dehydrogenase, long-chain	1.71
10	PREDICTED: similar to alpha enolase	-1.51
11	PREDICTED: similar to ATP synthase beta chain, mitochondrial precursor isoform 1	1.6
12	Annexin A4	1.9
13	PREDICTED: similar to eukaryotic translation initiation factor 4E isoform 1	-2.42
14	PREDICTED: similar to prohibitin	2.32
15	Lectin, galactose binding, soluble 1	1.56
16	Chain A, Importin Alpha, Mouse	-2.09
17	PREDICTED: similar to von Hippel-Lindau binding protein 1 isoform 2	1.51
18	PREDICTED: similar to chaperonin containing TCP1, subunit 2 isoform 2	1.74
19	Hypothetical protein LOC499689	1.53
20	MHC class II antigen beta chain	2.08
21	Chain A, Crystal Structure Of The Human Sh3 Binding Glutamic-Rich Protein Like	1.56

### Validation of results by Western Blotting Analysis

**Figure 4:**
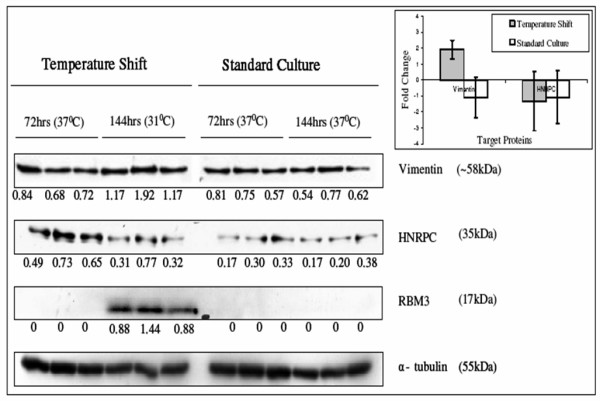
**Comparative Western blot analysis for HNRPC, vimentin and RBM3 proteins between 72 hrs and 144 hrs of culture in temperature shifted and standard culture.** α-tubulin is used as a loading control. The numbers below each band represents the intensities of bands after normalization to α-tubulin. The graph indicates the fold change, derived from densitometry analysis, of vimentin and HNRPC between 72 hrs and 144 hrs of culture. Error bars represent the standard deviation calculated from normalized intensities of bands from three biological replicate samples for vimentin and HNRPC.

Two proteins, vimentin and HNRPC, were selected from the list of differentially expressed identified proteins for validation by Western blotting. RBM3 is already known to be induced following shift to low temperature and was included as a positive control. α-Tubulin was used as a loading control. Western blot analysis reflected a similar pattern of expression to those observed using 2D-DIGE (Figure 4). Vimentin was 2.05 fold up regulated following temperature shift and 1.08 up regulated in standard culture from 2D-DIGE analysis. Densitometry analysis of Western blot data revealed that vimentin was 1.9 fold up regulated following temperature shift and 1.11 fold down regulated in standard culture. HNRPC was 1.52 fold down regulated following temperature shift and 1.26 fold down regulated in standard culture from 2D-DIGE analysis, while from densitometry analysis of western blot data, it was 1.33 fold down regulated at low temperature and 1.07 fold down regulated in standard culture.

## Discussion

The incorporation of a temperature reduction step is commonly employed during production cell culture in the biopharmaceutical industry. This temperature shift is used as a means of simultaneously inducing growth arrest and extending long term culture viability thus increasing recombinant protein productivity and yield [2-9]. To investigate the mechanisms regulating the effects of low temperature in CHO-K1 cells we employed 2D-DIGE followed by MALDI-ToF mass spectrometry.

When suspension-adapted CHO-K1 cells were cultured at 37°C for 72 hrs and then shifted to low temperature (31°C), cell growth was immediately arrested compared to cells maintained in standard cultures (37°C) (Figure 1A). Following the temperature shift, the cells were observed to maintain a steady viable cell density whereas the cells at 37°C began entering the death phase by 120 hrs of culture. While the viabilities of both cultures were comparable throughout the 144 hrs of culture this changed towards the end of the experiment for the cells maintained at 37°C (Figure 1). It is possible that the high percentage of serum (10%) in the culture medium protected both cultures from apoptotic cell death, therefore maintaining the high viabilities recorded. Serum is known to protect cells against apoptosis in suspension culture [22].

Following 2D-DIGE analysis to compare the differences in global protein expression at 72 hrs and 144 hrs of culture, 201 DE spots were observed in the temperature shifted cells whereas 404 DE spots were observed for the cells maintained in standard culture. It is apparent from this data that cells at the end of a temperature shifted batch culture are more similar to healthy exponentially growing cells than cells which have achieved late stationary/death phase at 37°C. Reduced cellular metabolism at low temperature could be responsible for the smaller number of differentially expressed proteins observed after temperature shift in comparison to the cells grown at 37°C. It has been observed that protein synthesis rates are reduced at low temperature due to a general inhibition of cap-dependent protein synthesis. However some proteins, *e.g*. RBM3, are translated under cold stress via an IRES mediated cap-independent mechanism [15,16]. This type of protein translation initiation requires both eukaryotic initiation factors (EIF2, EIF3, EIF4A, EIF4B, and EIF4F) and message-specific cellular IRES *trans*-acting factors (ITAFs) [23]. Eukaryotic translation initiation factor 4A, isoform 1 (EIF4A), an RNA helicase enzyme, is one of the critical translational initiation factors that are required in IRES mediated cap-independent protein translation [23]. EIF4A is 1.81 fold up regulated following temperature shift. This indicates that EIF4A may play an important role in facilitating translation in this system. EIF4A has been shown to be up regulated in G2 arrested CHO cells [24], which may also indicate a role for this protein in the observed growth arrest.

Of the 247 spots picked (127 up regulated and 120 down regulated at 144hrs), a total of 53 proteins were successfully identified (Additional file 1). Of these, 23 proteins (18 up regulated and 5 down regulated at 144 hrs) were specifically altered following temperature shift (Additional file 1). Two of these proteins (ALDH and DPYSL2) were present at more than one location in the gel indicating a low-temperature-specific protein modification. Conversely, 18 proteins were differentially regulated specifically in standard culture, of which one protein, ACTB, was present at two different positions in the gel. Grouping of the identified proteins by biological function indicated that proteins involved in metabolism were the dominant group affected in both temperature shifted and standard cultures (31 and 28% respectively), followed by structural proteins (14 and 21%). The indication that low temperature affects metabolism and structure is in keeping with other studies [12,14]. However, the number of proteins identified here is insufficient for a comprehensive analysis of the main biological functions affected in this system, as we may be observing a bias towards the identification of the more highly expressed metabolic and structural proteins within the cell such as GAPDH and vimentin.

The most obvious effect following reduction of culture temperature is the immediate decrease in growth observed. A number of growth-related proteins were identified as being differentially expressed in a temperature-specific manner, including HNRPC, RRN3 and LGALS1. HNRPC is a nuclear pre-mRNA binding protein that enhances the translation of c-myc mRNA by interacting with an IRES in the 5' UTR and thus impacting proliferation [25]. Silencing of HNRP C1/C2 protein expression by siRNA has also been shown to result in inhibition of cell proliferation [26]. These results are in keeping with our finding that HNRPC is 1.52 fold down regulated following reduction of the culture temperature. HNRPC also interacts with IRESs in the 5'UTR of 'upstream of N-Ras' (Unr) and affects cell proliferation through IRESs, including the PITSLRE IRES, which is activated at mitosis [26]. This is important because during mitosis, similar to low temperature culture inductions, protein synthesis is rapidly and severely repressed possibly due to inhibition of global cap-dependent translation [27]. The fact that HNRPC and EIF4A are both differentially expressed in this experiment clearly indicates the importance of IRES-based regulation of the translational machinery.

RRN3 protein levels were found to be 1.53 fold down regulated at the end of the temperature shifted culture which is unsurprising as its activity is known to be reduced in stationary cells [28]. LGALS1 (lectin, galactoside-binding, soluble, 1 or galectin 1) has been shown to act as a negative autocrine growth factor that regulates cell proliferation [29] which correlates with the 1.62 fold up regulation of LGALS1 observed in the temperature shifted samples and growth data.

Another common feature of cells that are cultured at reduced temperatures is that they display delayed apoptosis compared to cells cultured at 37°C [7,16]. It has been proposed that the reduction in apoptosis is as a result of the reduced cellular metabolism at low temperatures [7]. However, others have reported the induction of anti-apoptotic proteins (e.g. BCL-2) following culture at reduced temperature [30]. BCL-2 was not identified as a DE protein in this study but a number of apoptosis-associated proteins were identified including Importin-α, NADH dehydrogenase (ubiquinone) Fe-S protein 3 (NDUFS3) and Annexin A4. Importin-α, a nuclear import receptor, facilitates trafficking of the growth promoting Ras effector, RASSF5, into the nucleus [31]. Over expression of importin-α also results in significant increases in p21(waf1/cip1) transcript levels and apoptosis, implicating it in the nuclear import of p53 [32]. The temperature-specific 1.8 fold down regulation of importin-α levels therefore may have an impact on both proliferation and protection against apoptosis. Unlike importin-α, the other two apoptosis-related proteins Annexin A4 and NDUSF3 were both up regulated (1.95 and 1.51 fold respectively), and are positive regulators of apoptosis. Annexin A4 is selectively translocated from the nucleus to the cytosol during apoptosis and is recognized as an early marker of apoptotic cell death [33]. NDUFS3 is part of the mitochondrial complex I. Inhibition of NDUFS3 expression by siRNA has been shown to provide resistance to IFN-β/Retinoic Acid-induced apoptosis [34]. The data presented indicates that by the end of the temperature shifted culture the cells were beginning to initiate apoptosis. This is in agreement with other findings that despite the delay in apoptosis, once initiated, the rate and manner of cell death is similar to that observed in standard culture at 37°C [7].

By comparison, cells at 144 hrs in standard culture were already in early death phase (Figure 1). This is likely due to a variety of reasons including nutrient limitation, oxygen depletion and waste accumulation at 144 hrs in standard culture. A number of apoptosis related proteins, including prohibitin (PHB), Aldehyde dehydrogenase family 1, subfamily A1 (ALDH1A1) and alpha-enolase (ENO1) were found to be differentially regulated in this system (Additional file 1). Over expression of PHB inhibits cell proliferation by arresting cells in G0/G1 phase and has also been shown to increase cell survival [35]. ALDH1A1 is a member of aldehyde dehydrogenase family that can oxidize a wide variety of aldehydes (retinaldehyde, acetaldehyde, etc.) to the corresponding carboxylic acids (retinoic acid, acetic acid, etc.). Down regulation of ALDH1A1 has been correlated with the increased susceptibility of cells towards apoptosis [36]. ENO1 catalyses the formation of phosphoenolpyruvate from 2-phosphoglycerate. The over expression of ENO1 has been shown to inhibits cell growth and induce apoptosis in neuroblastoma cells [37]. PHB and ALDH1A1 were 1.66 and 1.64 fold down regulated respectively, while ENO1 was 1.51 fold up regulated at 144 hrs at 37°C in comparison to culture at 72 hrs. These observations are in keeping with the reduced viability observed at 144 hrs in standard culture in comparison to temperature shifted cultures.

A number of cytoskeletal proteins including vimentin, profilin, beta actin (ACTB) and capping protein (actin filament) muscle Z-line beta were differentially expressed following temperature shift and/or in standard culture (Additional file 1). Vimentin, profilin and beta-actin were also observed to be differentially expressed by Baik et. al. [12]. Vimentin is one of the major cytoskeletal proteins and its increased expression has previously been shown in the heat-resistant phenotypes of CHO cells [38]. Vimentin was 2.05 fold up regulated following temperature shift to 31°C and was unchanged at standard culture. Profilin is a small ubiquitous actin monomer sequestering protein that is required for normal actin polymerization in response to thermal stress [39], and was 1.69 fold up regulated following temperature shift. However profilin was unchanged at standard culture. ACTB is involved in many biological functions in cell such as regulating cell shape and growth. Increased expression of ACTB has been shown to provide resistance against apoptosis [40]. ACTB was 1.97 fold down regulated at 144 hrs at 37°C and was not significantly altered following temperature shift. CAPZB binds to one end of actin filaments, regulates actin polymerization including the number and length of actin filaments, and strengthens the actin cytoskeleton in the cytoplasm [41]. CAPZB was 1.5 fold down regulated at 144 hrs at 37°C, whereas no change was observed following temperature shift. Although the role of vimentin and profilin in regulating low temperature specific phenotypes is unclear at this point, the reduced expression of ACTB and CAPZB could possibly be associated with increased apoptosis in standard culture [40,41].

Cellular metabolism is generally reduced in low culture temperature [7] but this does not apply to every enzyme and pathway. GAPDH, a multi-functional glycolytic enzyme, was almost 2-fold up regulated following temperature shift. Similar results have been reported in other published data [12] (Table 1). In contrast metabolic enzymes involved in energy generation (PYP and ATP5B) were down regulated in dying cells (Additional file 1) which indicates reduced energy generation in standard culture at 37°C. This may be due to depletion of nutrients in the media at 144 hrs of culture.

Out of 53 identified proteins, 12 proteins (4 upregulated and 8 down regulated at 144 hrs compared to 72 hrs) were differentially expressed in both the temperature shifted cells and those maintained at 37°C (Table 2). In most cases the regulation between 72 hrs and 144 hrs was similar in the temperature shifted and standard cultures indicating that these are culture-dependent changes. As with the DE proteins identified from the temperature shift, these proteins cover a range of functions including stress response e.g. AHSA1. Of the 53 identified proteins, 21 were found to be differentially expressed between temperature shifted and non-shifted cultures at 144 hrs (Table 3).

Three proteins, ACTB, ALDH and DPYSL2, were identified from multiple spots. ACTB was present at two different pIs and molecular weights in the gel containing samples from standard culture. ALDH and DPYSL2 were identified at two different pIs on gels from the temperature shifted culture suggesting potential low temperature-induced post translational modifications. ALDH is a NAD(P)^+^-dependent enzyme that can protect against hyperoxia-induced cell death through reduction of ROS, activation of ERK/MAPK, and PI3K-Akt cell survival signalling pathways [42]. Analysis of the ALDH spots revealed 1.63 and 1.98 fold up regulation following temperature shift and no change in the cultures maintained at 37°C. The role of this protein in low temperature response is unknown. DPYSL2 promotes microtubule formation by binding to tubulin heterodimers [43]. Different variants, possibly phosphorylated, of DPYSL2 have been reported previously [44]. In this study, DPYSL2 was 2.58 fold up regulated following temperature shift whereas it was 1.97 fold up regulated in the cultures maintained at 37°C. However, the second spot identified as DPYSL2 was specifically 2.51 fold down regulated at 31°C. The functional association of DPYSL2 with low temperature response is unclear. In-silico phosphoproteome analysis predicted that both ALDH and DPYSL2 contain phosphorylation sites and hence the multiple spots may indicate that they are phosphorylated. A previous study also identified two proteins as being phosphorylated at tyrosine residues in CHO cells cultured at low temperature [2] suggesting that active cell signalling occurs in response to temperature shifts.

## Conclusion

In conclusion, the present study has identified a number of temperature-specific as well as temperature-independent changes in protein expression that occur during batch suspension culture. A number of the protein changes can be linked to the growth inhibition observed subsequent to temperature shift. Further studies targeting the proteins identified in this study using CHO-K1 cells producing a recombinant protein product will improve the understanding of the cold shock response and may be useful in identifying candidate targets to re-engineer CHO cells to produce recombinant therapeutic proteins more efficiently.

## Methods

### Cell Culture

Suspension-adapted CHO-K1 cells were used in this study. The culture medium consisted of ATCC medium (DMEM/F-12 Ham containing glutamine and sodium pyruvate; Sigma) supplemented with 10% fetal bovine serum (Sigma). Clearly serum concentration or use of serum-free medium could impact on protein expression profiles. Cells were maintained in 250 mL spinner vessels (Techne) at 60 rpm on spinner platforms in 37°C or 31°C incubators as appropriate. For batch culture experiments, exponentially growing cells were inoculated at 1 × 10^5 ^cells/mL into spinner vessels at a final volume of 100 mL. All cultures were gassed with compressed air (Air Products) each day for 1 min. Cell counts were taken every 24 hrs using a hemacytometer and the trypan blue exclusion method. For both temperature shifted and standard cultures, triplicate spinner vessels were taken for sampling following 72 and 144 hrs of culture.

### Sample Preparation and Protein Labelling for 2D-DIGE

Frozen cell pellets containing 2 × 10^7 ^cells were thawed and re-suspended in 400 μL of lysis buffer (7 M urea, 2 M thiourea, 30 mM Tris, 4% CHAPS, 5 mM magnesium acetate, pH 8.5), and then homogenised by carefully passing the samples through a 20 gauge needle 5 times. Samples were left on a shaker for 1 hr at room temp to allow extraction to take place, and then centrifuged at 14,000 rpm (or equivalent g) for 15 min at room temperature to remove insoluble material. The supernatant was removed and stored at -80°C until required for use. Protein concentration was determined using the thiourea-compatible Bradford protein assay (Bio-Rad).

For 2D-DIGE experiments, control and test cell lysates were labelled with 200 pmol of either Cy3 or Cy5 fluorescent dyes (GE Healthcare) for comparison on the same gel. Labelling reactions were performed on ice in the dark for 30 min and then quenched with a 50-fold molar excess of free lysine to dye for 10 min on ice. An internal standard containing a pool of all samples (both control and test) was labelled with Cy2 fluorescent dye, and this was used as a standard on all gels to aid image matching and cross-gel statistical analysis. The Cy3 and Cy5 labelling reactions (50 μg of each) from each lysate were mixed and run on the same gels with an equal amount (i.e. 50 μg per gel) of Cy2-labelled standard. Technical duplicates of 3 independent biological replicates resulted in 36 fluorescent dye gel images being analysed in this study.

### Protein Separation by 2D-DIGE

18 cm IPG 4–7 strips (GE Healthcare) were rehydrated overnight in buffer (7 M urea, 2 M thiourea, 4% CHAPS, 0.5% IPG buffer, 50 mM DTT) according to manufacturer's guidelines. Isoelectric focussing was performed using the IPGphor (GE Healthcare) for a total of 50 kV/hrs at 20°C with the resistance set at 50 mA per strip. After IEF the strips were equilibrated for 15 min in 50 mM Tris-HCl pH 8.8, 6 M urea, 30% (v/v) glycerol, 1% (w/v) SDS supplemented with 65 mM DTT, followed by a second 15 min equilibration in the same buffer containing 240 mM iodoacetamide instead of DTT. The equilibrated strips were then transferred onto large format 12.5% acrylamide gels poured between low fluorescence glass plates, and then overlaid with 0.5% agarose solution containing bromophenol blue. The gels were placed in the Ettan Dalt 12 electrophoresis unit (GE Healthcare) and run at 1.5 W/gel at 10°C overnight until the dye-front had reached ~1 cm from the bottom of the gel.

### Image Acquisition and Data Analysis

All of the gels were scanned using the Typhoon 9400 Variable Mode Imager (GE Heathcare) to generate gel images at the appropriate excitation and emission wavelengths from the Cy2, Cy3 and Cy5 labelled samples. The resultant gel images were cropped using the ImageQuant software tool and imported into Decyder 6.5 software. The Biological Variation Analysis (BVA) module of Decyder 6.5 was used to compare the control versus test samples to generate lists of differentially expressed proteins. For statistical purposes, a cut-off of 1.5 fold up/down regulated with a t-test score < 0.05 was used.

### Spot Digestion

Preparative gels containing 400 μg of protein were fixed and then post-stained with colloidal coomassie blue stain (Sigma). The gels were scanned and images imported into ImageMaster Platinum software (GE Healthcare) and matched to the Master gel image generated from the DIGE experiment. Spots of interest were selected and a pick list was generated and imported into the software of the Ettan Spot Picker robot (GE Healthcare). Gel plugs were placed into presiliconised microtitre plates and stored at 4°C until digestion. Tryptic digestions were performed using the Ettan Digestor robot (GE Healthcare). Excess liquid was removed from each plug, and washed for 3 cycles of 20 min using 50 mM NH_4_HCO_3 _in 50% methanol solution. The plugs were then washed for 2 cycles of 15 min using 70% ACN and left to air dry for 1 hour. Lyophilised sequencing grade tryspin (Promega) was reconstituted with 50 mM acetic acid as a stock solution and then diluted to a working solution with 40 mM NH_4_HCO_3 _in 10% ACN solution, to a concentration of 12.5 ng trypsin per μL. Samples were digested at 37°C overnight and were then extracted twice with 50% ACN and 0.1% TFA solution for 20 min. each. All extracts were pooled and concentrated by SpeedVac (Thermo Scientific) for 40 min.

### MALDI-ToF analysis of peptides

For MALDI-ToF analysis, the peptide samples were desalted and concentrated using C-18 Zip-Tips (Millipore). Each tip was wetted firstly using 100% ACN, then with 50% ACN solution with 0.1% trifluoroacetic acid (TFA), and then equilibrated with 0.1% TFA in LC-MS grade water (Sigma). The bound peptides were eluted directly onto MALDI-ToF MS target slides with 5 mg/mL of α-cyano-4-hydroxycinnamic acid (Laser Biolabs) in 50% acetonitrile/0.1% TFA (v/v) solution. An internal sample mix, Pep4 (Laser Biolabs) was also spotted onto target slides and used as an internal calibrant.

Mass spectra were recorded in the positive ion, reflectron mode using the Ettan MALDI-ToF Pro mass spectrometer (GE Healthcare) equipped with delayed extraction and a standard nitrogen laser (337 nm). The spectra were internally calibrated with trypsin enzyme autolysis peptide peaks at m/z 842.51 and m/z 2211.10, and Pep4 mix when possible. Spectra were acquired by selective accumulation of 250 individual laser shots and processed using Ettan MALDI evaluation software. Known contaminant peaks were removed from the resulting mass spectra and remaining sample-related peaks were used for database searching. The artificial modifications of peptides (carbamidomethylation of cysteines and partial oxidation of methionines) were also considered. Protein identification was achieved using Ettan MALDI-ToF Pro evaluation software (GE Healthcare) incorporating Profound database search engine for peptide mass fingerprints. The sequence database searched was NCBI-nr database (2007/12/02) using subset species, Mammalian, Rodentia and *Homo sapiens*.

### Western Blot Analysis

10–40 μg protein were separated on 10% SDS polyacrylamide gels and transferred onto Hybond ECL nitrocellulose membranes (GE) using the semi-dry transfer method. Membranes were blocked with 1–5% Marvel-PBS for 1 hr at room temperature and probed overnight at 4°C with mouse anti-hnRNP C (Sigma), goat anti-vimentin (Sigma), rabbit anti-RBM3 (kindly supplied by Peter W. Vanderklish, Scripps Research Institute) and mouse anti-tubulin (Sigma) antibodies. Blots were then washed and incubated with the appropriate peroxidase-labelled secondary antibodies (anti-mouse secondary (DakoCytomation), anti-goat secondary (Sigma), and anti-rabbit secondary (DakoCytomation)). After washing, bands were visualized by the enhanced chemiluminescence (ECL) method (GE Healthcare). α-Tubulin was used as a loading control.

## Authors' contributions

NK carried out the cell culture, proteomics work, interpretation of results and manuscript preparation of this study. PG participated in the experimental design of the study, cell culture, interpretation of results and manuscript preparation. PM participated in the experimental design of the proteomics part of the study, statistical analysis of the 2D-DIGE gels and manuscript preparation. MH participated in the identification of differentially expressed proteins by mass spectrometry. MC conceived the study, participated in its design and coordination. All authors read and approved the final manuscript.

## Supplementary Material

Additional file 1 MALDI-ToF MS identification of proteins that were differentially expressed at 144 hrs compared to 72 hrs using either temperature shifted or standard culture. Click here for file
