# Supplementary material for: Differential protein expression following low temperature culture of suspension CHO-K1 cells
Source: BMC Biotechnol. 2008 Apr 22;8:42. doi: 10.1186/1472-6750-8-42 (PMC2386802; doi:10.1186/1472-6750-8-42)
Supplement: Additional file 1 — MALDI-ToF MS identification of proteins that were differentially expressed at 144 hrs compared to 72 hrs using either temperature shifted or standard culture. [file 1472-6750-8-42-S1.doc]

**Additional file 1**. MALDI-ToF MS identification of proteins that were differentially expressed at 144hrs compared to 72hrs using either temperature shifted or standard culture.

| **Number** | **Protein ID** | **Protein Description** | **Abbreviation** | **Expectation value^a^** | **Peptides Matched for Identification** | **% Coverage** | **Temperature Shift** | | **Standard Culture** | |
| --- | --- | --- | --- | --- | --- | --- | --- | --- | --- | --- |
|  |  |  |  |  |  |  | Fold Change^b^ | t-test score | Fold Change^b^ | t-test score |
| **Structural** | | | | | | | | | | |
| 1 | gi|1407651 | LIM and SH3 domain protein Lasp-1 | LASP1 | 0.005 | 6 | 33.2 | 2.06 | 7.20E-09 | NDE | NDE |
| 2 | gi|2078001 | Vimentin | VIM | 0 | 19 | 44.6 | 2.05 | 3.70E-07 | NDE | NDE |
| 3 | gi|55391513 | Calponin 3, acidic | CNN3 | 0.008 | 6 | 19.7 | -1.96 | 7.20E-09 | -3.37 | 2.80E-10 |
| 4 | gi|55621900 | PREDICTED: similar to profilin 2 isoform b | PFN2 | 0 | 5 | 39.3 | 1.69 | 1.00E-03 | NDE | NDE |
| 5 | gi|2624850 | Chain A, Structure Of Bovine Beta-Actin-Profilin Complex (1HLUA) | --- | 0.002 | 10 | 44.8 | -1.59 | 3.60E-08 | -1.53 | 1.20E-11 |
| 6 | gi|16304154 | Beta actin | ACTB | 0.003 | 5 | 17.9 | NDE | NDE | -1.97 | 4.50E-09 |
| 7^c^ | gi|60389477 | Beta actin | ACTB | 0.001 | 8 | 30.7 | NDE | NDE | -1.74 | 4.20E-10 |
| 8 | gi|54695812 | Capping protein (actin filament) muscle Z-line, beta | CAPZB | 0.005 | 8 | 26.5 | NDE | NDE | -1.5 | 1.90E-05 |
| 9 | gi|224839 | Tubulin T beta15 | TUBB2B | 0.009 | 7 | 18 | NDE | NDE | -2.02 | 2.00E-08 |
| **Metabolism** | | | | | | | | | | |
| 1 | gi|16073616 | Aldehyde dehydrogenase | ALDH | 0 | 6 | 18.7 | 1.98 | 5.50E-08 | NDE | NDE |
| 2^d^ | gi|16073616 | Aldehyde dehydrogenase | ALDH | 0.004 | 9 | 22.1 | 1.63 | 5.80E-06 | NDE | NDE |
| 3 | gi|28386049 | Aldehyde dehydrogenase family 1, subfamily A1 | ALDH1A1 | 0.001 | 8 | 20 | NDE | NDE | -1.64 | 0.00011 |
| 4 | gi|2897818 | Huntingtin interacting protein-2 | HIP2 | 0.006 | 5 | 28.5 | 1.97 | 1.40E-06 | NDE | NDE |
| 5 | gi|56783068 | Glyceraldehyde-3-phosphate dehydrogenase | GAPDH | 0.004 | 5 | 22.3 | 1.8 | 4.00E-07 | NDE | NDE |
| 6 | gi|206428 | Phosphoribosylphosphate synthetase (PRPS2) precursor | PRPS2 | 0 | 5 | 21.7 | 1.53 | 1.6E-06 | NDE | NDE |
| 7 | gi|55635845 | PREDICTED: similar to NADH dehydrogenase (ubiquinone) Fe-S protein 3, 30kDa (NADH-coenzyme Q reductase) | NDUFS3 | 0.007 | 7 | 25.5 | 1.51 | 2.30E-07 | NDE | NDE |
| 8 | gi|57110953 | PREDICTED: similar to NADH dehydrogenase (ubiquinone) Fe-S protein 1, 75kDa precursor | NDUFS1 | 0.008 | 8 | 12.2 | 2 | 8.40E-08 | 1.59 | 3.80E-06 |
| 9 | gi|76611941 | PREDICTED: similar to UDP-N-acetylhexosamine pyrophosphorylase (Antigen X) | AGX | 0.002 | 6 | 12.8 | -1.93 | 1.70E-06 | NDE | NDE |
| 10 | gi|31982520 | Acetyl-Coenzyme A dehydrogenase, long-chain | ACADL | 0.005 | 10 | 24.2 | 1.88 | 7.8E-008 | NDE | NDE |
| 11 | gi|6679891 | Alpha glucosidase 2, alpha neutral subunit | GANAB | 0.009 | 12 | 11.9 | 2.19 | 5.60E-09 | NDE | NDE |
| 12 | gi|5759173 | Succinate dehydrogenase flavoprotein subunit | SDHA | 0.003 | 9 | 19.3 | 1.7 | 5.90E-08 | 1.87 | 6.90E-10 |
| 13 | gi|71059735 | Pyrophosphatase | PYP | 0.001 | 8 | 33.9 | NDE | NDE | -1.58 | 3.60E-10 |
| 14 | gi|38512111 | Triosephosphate isomerase 1 | TPI1 | 0.001 | 8 | 29.8 | NDE | NDE | 1.51 | 5.60E-08 |
| 15 | gi|74007151 | PREDICTED: similar to alpha enolase | ENO1 | 0.007 | 9 | 30.3 | NDE | NDE | 1.51 | 4.80E-07 |
| 16 | gi|73968432 | PREDICTED: similar to ATP synthase beta chain, mitochondrial precursor isoform 1 | ATP5B | 0 | 10 | 24.9 | NDE | NDE | -2.28 | 1.60E-06 |
| 17 | gi|25320034 | Adenosine kinase | ADK | 0.006 | 6 | 16.1 | NDE | NDE | 1.78 | 3.50E-12 |
| **Differentiation** | | | | | | | | | | |
| 1 | gi|57105264 | PREDICTED: similar to Dihydropyrimidinase related protein-2 | DPYSL2 | 0.007 | 15 | 11.2 | 2.58 | 5.90E-08 | 1.97 | 3.50E-10 |
| 2^e^ | gi|3122018 | Dihydropyrimidinase-related protein 2 | DPYSL2 | 0.009 | 5 | 10.5 | -2.51 | 5.50E-07 | NDE | NDE |
| **Signal Transduction** | | | | | | | | | | |
| 1 | gi|55742832 | Annexin A4 | ANXA4 | 0.001 | 11 | 35.4 | 1.95 | 7.20E-09 | NDE | NDE |
| 2 | gi|28876 | Growth factor receptor-bound protein 2 | GRB2 | 0.002 | 5 | 27.5 | NDE | NDE | -1.72 | 2.40E-09 |
| **Translation** | | | | | | | | | | |
| 1 | gi|66910561 | Heterogeneous nuclear ribonucleoprotein C | HNRPC | 0 | 8 | 32.9 | -1.52 | 6.9E-07 | NDE | NDE |
| 2 | gi|20987331 | Eukaryotic translation initiation factor 3, subunit I | EIF3I | 0.008 | 5 | 20 | -1.72 | 1.70E-08 | -2 | 7.50E-11 |
| 3 | gi|73978223 | PREDICTED: similar to eukaryotic translation initiation factor 4A, isoform 1 | EIF4A | 0 | 6 | 21.7 | 1.81 | 7.30E-07 | NDE | NDE |
| 4 | gi|74001995 | PREDICTED: similar to eukaryotic translation initiation factor 4E isoform 1 | EIF4E | 0.005 | 6 | 30.4 | NDE | NDE | 1.67 | 0.0013 |
| 5 | gi|109296 | Eukaryotic translation initiation factor 5A | EIF5A | 0.001 | 6 | 34.4 | -1.81 | 1.30E-07 | -2.07 | 7.50E-08 |
| 6 | gi|56967054 | Chain A, Tu translation elongation factor, mitochondrial | TUFM | 0.003 | 7 | 23.5 | NDE | NDE | 1.56 | 4.60E-07 |
| 7 | gi|73989786 | 40S ribosomal protein SA (p40) [Canis familiaris] | LOC480358 | 0 | 7 | 41.3 | NDE | NDE | -1.9 | 2.40E-06 |
| **Transcription** | | | | | | | | | | |
| 1 | gi|21706613 | Rrn3 protein | RRN3 | 0.001 | 3 | 5.7 | -1.53 | 7.40E-10 | NDE | NDE |
| 2 | gi|73966295 | PREDICTED: similar to prohibitin | PHB | 0.002 | 4 | 25.2 | NDE | NDE | -1.66 | 4.30E-10 |
| **Cell adhesion** | | | | | | | | | | |
| 1 | gi|12805209 | Lectin, galactose binding, soluble 1 | LGALS1 | 0.007 | 4 | 32.6 | 1.62 | 5.70E-05 | NDE | NDE |
| **Stress Response** | | | | | | | | | | |
| 1 | gi|73963752 | PREDICTED: similar to Activator of 90 kDa heat shock protein ATPase homolog 1 | AHSA1 | 0.003 | 8 | 15.5 | -1.69 | 9.70E-09 | -1.73 | 4.60E-10 |
| **Protein binding** | | | | | | | | | | |
| 1 | gi|78187979 | Thioredoxin-like 2 | TXNL2 | 0.009 | 7 | 19.3 | -1.8 | 4.60E-08 | -1.8 | 7.90E-09 |
| 2 | gi|5542272 | Chain A, Importin Alpha, Mouse | Importin-α | 0.007 | 5 | 13.5 | -1.8 | 8.80E-05 | NDE | NDE |
| **Secretory pathway** | | | | | | | | | | |
| 1 | gi|1698802 | Menkes disease gene product | ATP7A | 0.005 | 6 | 6.2 | 1.79 | 9.30E-07 | 1.6 | 9.00E-10 |
| **Protein Folding** | | | | | | | | | | |
| 1 | gi|13097417 | FK506 binding protein 4 | FKBP4 | 0.01 | 7 | 13.1 | -2.5 | 7.40E-10 | -2.43 | 1.50E-10 |
| 2 | gi|73954621 | PREDICTED: similar to heat shock protein 8 isoform 3 | HSPA8 | 0.003 | 7 | 15.7 | -1.9 | 5.60E-09 | -2.09 | 2.20E-09 |
| 3 | gi|74008872 | PREDICTED: similar to von Hippel-Lindau binding protein 1 isoform 2 | VBP1 | 0.007 | 7 | 26.9 | NDE | NDE | -1.8 | 1.20E-10 |
| 4 | gi|73968675 | PREDICTED: similar to chaperonin containing TCP1, subunit 2 isoform 2 | CCT2 | 0.001 | 10 | 29.6 | NDE | NDE | -1.64 | 2.60E-08 |
| **Miscellaneous** | | | | | | | | | | |
| 1 | gi|66730313 | Hypothetical protein LOC499689 | --- | 0.004 | 6 | 23.4 | 2.2 | 2.80E-08 | NDE | NDE |
| 2 | gi|13161222 | MHC class II antigen beta chain | MHC II β | 0.007 | 7 | 62.9 | 1.87 | 2.70E-08 | NDE | NDE |
| 3 | gi|75765278 | Chain A, Crystal Structure Of The Human Sh3 Binding Glutamic-Rich Protein Like | SH3BGRL | 0 | 8 | 74.4 | 1.7 | 1.80E-07 | NDE | NDE |
| 4 | gi|73963786 | PREDICTED: similar to Protein C14orf166 | --- | 0.002 | 8 | 24.6 | 1.5 | 1.80E-07 | NDE | NDE |
| 5 | gi|12841560 | Unnamed protein product | --- | 0.006 | 5 | 43.8 | NDE | NDE | -1.54 | 1.60E-08 |

**Legends**

^a^ Expectation value is an approximation of “the probability that a certain protein candidate is a random hit”.

^b^ Fold change was calculated by DeCyder by dividing the spot density at 144hrs by that at 72hrs.

c/d/^e^ represents the proteins identified at multiple spots.

NDE: proteins that are **N**ot **D**ifferentially **E**xpressed at cut-off criteria of at least 1.5 fold up/down regulated with a t-test score ≤0.05.

‘---‘: Not specified
